# Ion-Molecule Reactions and Chemical Composition of Emanated from Herculane Spa Geothermal Sources

**DOI:** 10.3390/ijms9061024

**Published:** 2008-06-20

**Authors:** Constantin Cosma, Ioan Suciu, Lorentz Jäntschi, Sorana D. Bolboacă

**Affiliations:** 1 Babeş Bolyai University, Faculty of Environmental Science, 1 M. Kogălniceanu, 400084 Cluj-Napoca, Romania; E-mail(s): cosmac@enviro.ubbcluj.ro; siiioan@yahoo.com; 2 Technical University of Cluj-Napoca, 103–105 Muncii Bvd, 400641 Cluj-Napoca, Romania; E-mail: lori@academicdirect.org; 3 Iuliu Haţieganu University of Medicine and Pharmacy Cluj-Napoca, Department of Medical Informatics and Biostatistics, 6 Louis Pasteur, 400349 Cluj-Napoca, Romania

**Keywords:** Ion-molecule reactions, Radioactivity, Methane, Methane upper homologues

## Abstract

The paper presents a chemical composition analysis of the gases emanated from geothermal sources in the Herculane Spa area (Romania). The upper homologues of methane have been identified in these gases. An ion-molecule reaction mechanism could be implicated in the formation of the upper homologues of methane. The CH_4_^+^ ions that appear under the action of radiation are the starting point of these reactions. The presence of hydrogen in the emanated gases may be also a result of these reactions.

## 1. Introduction

Herculane Spa (*Aqua Hercilis*, lat.) is a town in Caraş -Severin County, Romania. It is situated at an altitude of 165 m in the Cerna River valley, between Mehedinţi (to the east) and Cerna Mountains (to the west). The Romans discovered the curative value of the thermal mineral waters found in Herculane Spa almost 2,000 years ago. It is one of the oldest health resorts in Romania and one of the oldest in the world, being renowned for its eighteen thermal mineral water springs. Three different types of thermal mineral water with different hydrogeological origins are identified in this area [1]: (a) cold water, (b) hot water (deep cold waters that are thermalized), and (c) thermal mineral water.

The thermal mineral waters from Herculane Spa are used in treatment of rheumatic [2], neurological [3], gynaecological [4] and dermatological diseases [5], or for rehabilitation of posttraumatic patients [6]. The geothermal waters from Herculane Spa have their origin in granites and sedimentary rocks (marls and limestone), the latter forming the impermeable roof of the hydrothermal deposit [7]. A big and deep fissure following the Cerna canyon is present and many transversal fissures exist. The thermal water springs are thus accompanied by large amounts of gases such as nitrogen and methane (which are the main components of emanated gases), helium and radon. Measurements in the Herculane Spa region have shown high helium (5–10 ml/L [8,9]) and radon (7.4 MBq·m^−3^) [10] concentrations.

The water of these deposits is permanently regenerated from three components [11]: a) uphill infiltration water from the Cerna Valley, b) deposit type water, and c) boiling hot deep water.

The methane system is one of the most investigated ion-molecule systems [12–14]. The presence of the upper homologues of methane (C_2_H_6_, C_3_H_8_, etc.) in gases near geothermal waters springs can be explained by the existence of the ion-molecule reactions. Theses reactions have as a starting point the CH_4_^+^ ions that are formed after the impact with α, β radiation [10].

The existence of a high radioactivity level is due to the presence of uranium in the Cerna River valley area, which is confirmed by high radon activity and large helium concentrations in the studied gases (the source of nuclear radiations), which could be the factor responsible for initiating the ion-molecule reactions.

The presence of high radon activity as well as the presence of hydrogen at relatively high quantities leads to the suggestion that the apparition of the upper homologues of methane is a result of ion-molecule reactions. Radon activity was not previously identified as being at high levels in the Ghizela’s Cross Spa and Mehadia coal mine [6]. The aim of the present research was to investigate and analyze the chemical composition of the gases emanated by the geothermal sources in Herculane Spa area, in order to explain the presence of high concentrations of helium.

## 2. Experimental Section

### 2.1. Sampling of the Herculane Spa Area

Twenty-four mineral waters sources are present in Cerna Canyon, in the Herculane Spa area (Băile Herculane, © 2000–2008, viewed 01 June 2008, available from: http://www.baile-herculane.ro/; Herculane’s Spa Map (in Romanian), viewed 03 May 2008, Available from: http://www.ici.ro/romania/en/turism/b_herculane1.html). The spas from Herculane Spa area display high radioactivity [7] and are comparable with the spas from Vichy (thermal springs, 46°07′40″N and 3°25′36″E, Allier - France) and Mont-Dore-les-Bains (thermal springs, 45°35′N and 2°49′E, Puy-de-Dôme - France). Almost sixty-seven percent of the spas are springs (sixteen out of twenty-four) and the other spas are drillings. The Herculane spas produce a total water flow of 65000 L/24h. Due to the small debit, some of mineral water sources are not used any more.

**Figure 1.**
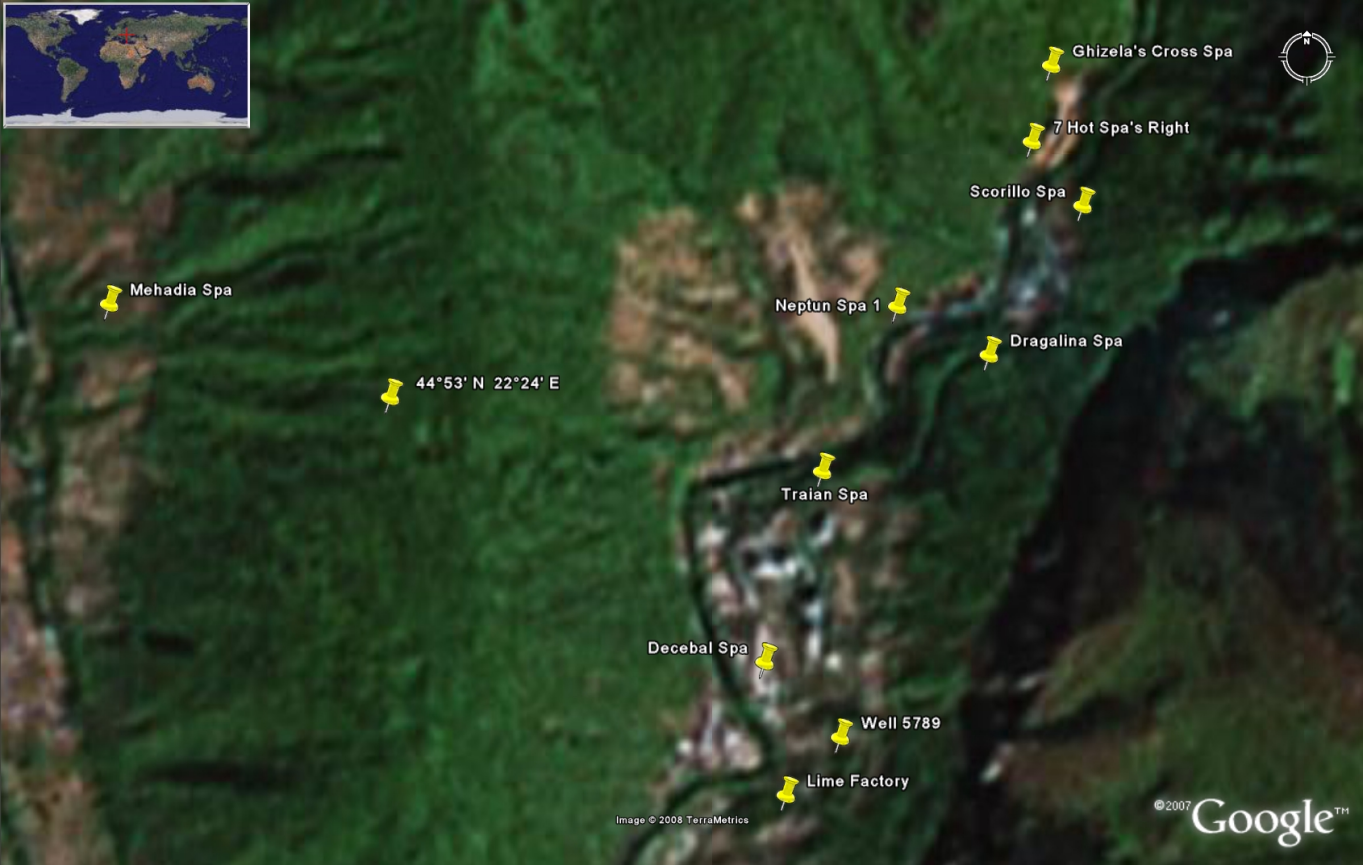
Geothermal sources and gas measurement sites in the Herculane Spa area.

Nine geothermal sources in Herculane Spa and the Mehadia coal mine were investigated (see Figure 1 and Table 1). The main criterion in the selection of the sources was a high debit of the emanated gases. The soil is of granite on the surface in the Ghizela’s Cross Spa, Seven Hot Spas and Scorillo Spa areas. Granite covered by sedimentary rock soil is present otherwise. Radon flux is observed near the following spas: Ghizela’s Cross, Scorillo, and Neptun 1.

### 2.2. Methods used of Gases Collection

**Figure 2.**
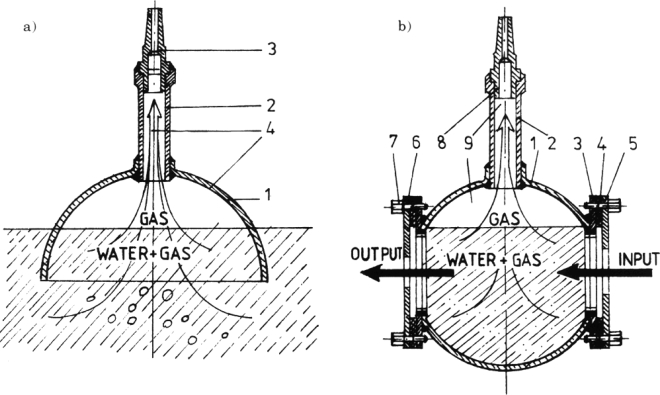
(a) Device for the sampling of the emanated gases from springs (1= metallic semi-sphere; 2 = metallic pipe; 3 = joint; 4 = gas flow). (b) Device for the sampling of the emanated gases from drilling (1= metallic sphere; 2 = metallic pipe; 3 = metallic fixed flange; 4 = gasket; 5,6 = metallic interchangeable flanges; 7 = screws for fixing flanges; 8 = joint; 9 = gas flow).

The water springs and drillings carry high quantities of gases to the surface as gas bubbles. The gases bubbles are always in excess, compared with the dissolved gases [15]. Two special devices were designed, developed and used for sampling the gases, one for the springs and the other for drilling sources of thermal water (see Figure 2).

Some 6–7 km west of Herculane Spa, there is an important coal deposit organized on many levels, the last of these being deep coal shells (about 1,000–1,200 m). This coal deposit could be the methane source for the gases from the Herculane Spa geothermal sources. As it is well known, coal can contain large quantities of gases (up to 6–8 NTP m^3^/1,000 kg). This was the reason for including the Mehadia coal mine in the analysis.

### 2.3. Determination of Gases Composition

The samples of gases from Herculane Spa were collected in October 1986. The determinations of the gases composition were done using a Dempster mass-spectrometer. Three measurements were performed at Babeş Bolyai University for each sample.

Binary mixtures of different gases (N_2_ + CH_4_; N_2_ + Ar; N_2_ + C_2_H_6_, CH_4_ + Ar, etc.) were used as standards. The determination of a given gas concentration was done by comparing the mass peaks of standard with the mass peak of the sample [5].

## 3. Results and Discussion

The concentrations of the main constituents of the emanated gases from the investigated geothermal sources in Herculane Spa area and the gas composition of the gases extracted from the coal originating from Mehadia mine are presented in Table 2. The values represent the average of three determinations. The relative deviation between the measurements was of 5%.

The analyzed places could be classified in three groups according to the composition of emanated gases. First group contains a single thermal mineral drilling water source (Ghizela’s Cross Spa, see Table 2). The gases identified in this first group could represent atmospheric gases that were processed underground, as suggested by a high oxygen content (14.73 %). The oxygen concentration decreased by almost 7% due to its reactivity in the underground route. Four out of eight investigated gases (CH_4_, C_2_H_6_, H_2_S, and H_2_) were not identified in this source. The inert gases N_2_ and Ar were attached to the composition of emanated gases.

The second group contains one drilling and one spring source (Scorillo and 7 Hot Spa’s Right, see Table 2). A high content of nitrogen (95.12%, 96.28% respectively), and a lower concentration of methane and ethane (less than 3%) were measured for these sources. Contrary to the first source, oxygen was measured at a very low concentration (0.12% compared with 14.73% in the first group).

**Figure 3.**
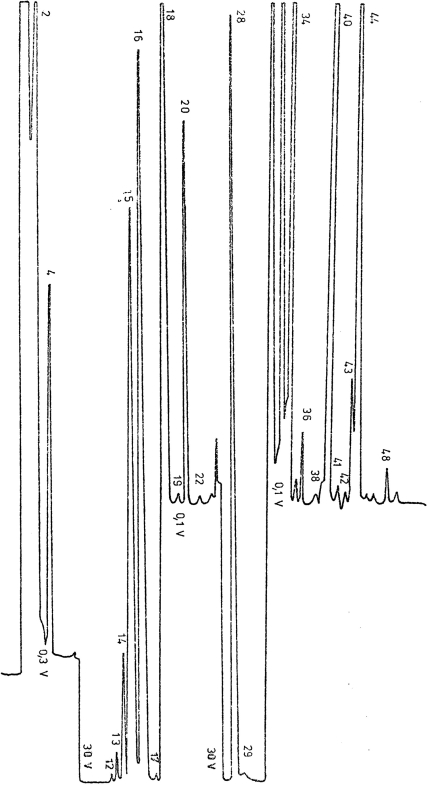
Mass spectrum: Neptun 1 Spa sample.

**Figure 4.**
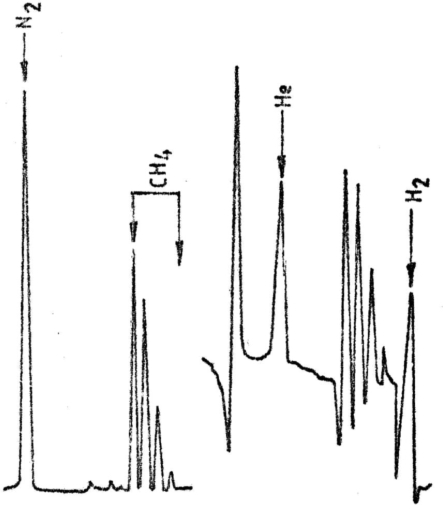
Mass spectrum: Lime Factory.

The third group comprises of sources in which the main constituent was identified as being methane. This group contains six sources (Neptun 1 Spa, Dragalina Spa, Decebal Spa, Well 5789, Traian Spa, and Lime Factory). A mass spectrum obtained from analysis a sample from Neptun 1 Spa is presented in Figure 3. The mass spectrum obtained for a Lime Factory sample is presented in Figure 4.

The concentration of methane varied from 59.51% (Lime Factory) to 70.88% (Decebal Spa). The ethane concentration reaches up to 0.84 % in this group. A high concentration of helium has been previously reported from the sources of this group, the concentration of helium being the highest concentration in Romania [16]. Generally, the helium content follows the methane concentration. Lower concentrations of oxygen were identified in this group (with values from 0.15% found in Lime Factory to 0.02% found at Neptun and Well 5789 Spas). The hydrogen content was also high (up to 2%).

The last line of Table 2 (No. 10) presents the gas composition corresponding to the carbon-nitrogen gases extracted from Mehadia coal mine. The main components in the Mehadia coal mine are CO_2_ (79.63%), CH_4_ (12.01%), and N_2_ (8.05%). The concentrations of these gases are different from the others.

The main difference in the gas composition of the identified groups was in the level of methane, which was detected at high concentrations in the third group, compared with first and second ones. The low oxygen concentration in the second and third groups revealed the different origins of the gases in the investigated sources.

The atmospheric air geochemically processed by an underground route was slightly enriched with methane (migrated from the Herculane Spa) in the case of the first as well as the second group.

The gases identified in the third group of thermal mineral water sources are a mixture, in which the main component is the methane arising from the gases accumulated in the coal pores of Mehadia deep level deposit.

The presence of helium in these gases [5] and the classification of the geographical area as a high radioactivity zone [17] due to Cerna granites rocks sustain the following reaction mechanisms leading to C_2_H_6_ and upper homologues. The starting point is the CH_4_^+^ ion, which appears as a consequence of uranium-radon (and its descendants) α-attack.

The ion-molecule reactions of methane [18,19] are:

(1) $${{\text{CH}}_{4}}^{+}+\;{\text{CH}}_{4}\to {{\text{CH}}_{5}}^{+}+\;{\text{CH}}_{3}$$

(2) $${{\text{CH}}_{4}}^{+}+\;{\text{CH}}_{4}\to {{\text{C}}_{2}{\text{H}}_{4}}^{+}+\;2{\text{H}}_{2}$$

(3) $${{\text{CH}}_{4}}^{+}+\;{\text{CH}}_{4}\to {{\text{C}}_{2}{\text{H}}_{6}}^{+}+\;{\text{H}}_{2}$$

The CH_3_^+^, CH_2_^+^ and CH^+^ ions, forming the methane mass spectrum in the electron impact sources (α+β radiation in our case) can give the following reactions:

(4) $${{\text{CH}}_{3}}^{+}+\;{\text{CH}}_{4}\to {{\text{C}}_{2}{\text{H}}_{5}}^{+}+\;{\text{H}}_{2}$$

(5) $${{\text{CH}}_{3}}^{+}+\;{\text{CH}}_{4}\to {{\text{C}}_{2}{\text{H}}_{3}}^{+}+\;2{\text{H}}_{2}$$

(6) $${{\text{CH}}_{3}}^{+}+\;{\text{CH}}_{4}\to {{\text{C}}_{2}{\text{H}}_{4}}^{+}+\;{\text{H}}_{2}$$

(7) $${{\text{CH}}_{2}}^{+}+\;{\text{CH}}_{4}\to {{\text{C}}_{2}{\text{H}}_{3}}^{+}+\;{\text{H}}_{2}+\text{H}$$

(8) $${{\text{CH}}_{2}}^{+}+\;{\text{CH}}_{4}\to {{\text{C}}_{2}{\text{H}}_{4}}^{+}+\;{\text{H}}_{2}$$

(9) $${{\text{CH}}_{2}}^{+}+\;{\text{CH}}_{4}\to {{\text{C}}_{2}{\text{H}}_{2}}^{+}+\;2{\text{H}}_{2}$$

(10) $${\text{CH}}^{+}+{\text{CH}}_{4}\to {{\text{C}}_{2}{\text{H}}_{2}}^{+}+\;{\text{H}}_{2}+\text{H}$$

The secondary ions that appeared in reactions from Eq(4) to Eq(10) can subsequently give the following reactions:

(11) $${{\text{C}}_{2}{\text{H}}_{3}}^{+}+\;{\text{CH}}_{4}\to {{\text{C}}_{2}{\text{H}}_{4}}^{+}+\;{\text{CH}}_{3}$$

(12) $${{\text{C}}_{2}{\text{H}}_{3}}^{+}+\;{\text{CH}}_{4}\to {{\text{C}}_{3}{\text{H}}_{3}}^{+}+\;{\text{H}}_{2}+2\text{H}$$

(13) $${{\text{C}}_{2}{\text{H}}_{3}}^{+}+\;{\text{CH}}_{4}\to {{\text{C}}_{3}{\text{H}}_{4}}^{+}+\;{\text{H}}_{2}+\text{H}$$

(14) $${{\text{C}}_{2}{\text{H}}_{3}}^{+}+\;{\text{CH}}_{4}\to {{\text{C}}_{3}{\text{H}}_{5}}^{+}+\;2\text{H}$$

(15) $${{\text{C}}_{2}{\text{H}}_{3}}^{+}+\;{\text{CH}}_{4}\to {{\text{C}}_{3}{\text{H}}_{5}}^{+}+\;{\text{H}}_{\text{2}}$$

(16) $${{\text{C}}_{2}{\text{H}}_{3}}^{+}+\;{\text{CH}}_{4}\to {{\text{C}}_{3}{\text{H}}_{6}}^{+}+\;\text{H}$$

(17) $${{\text{C}}_{2}{\text{H}}_{3}}^{+}+\;{\text{CH}}_{4}\to {{\text{C}}_{3}{\text{H}}_{7}}^{+}+\;\text{H}$$

The cross section of these ion-molecule reactions is a function of the kinetic energy of molecular ions that increases when kinetic energy decreases [20]. At very small kinetic energies (thermal energy range), the reaction from Eq(17) is likely to produce propane directly:

(18) $${\text{C}}_{\text{2}}{{\text{H}}_{4}}^{+}+\;{\text{CH}}_{4}\to {\text{C}}_{3}{{\text{H}}_{8}}^{+}$$

The reactions presented in Eq(1) – Eq(18), occurring on a thermal energy range and initiated by CH_4_^+^, CH_3_^+^, CH_2_^+^, CH^+^ ions, are attributable to nuclear radiation (α and β). They can explain the appearance of upper methane homologues and the presence of hydrogen in the gases from the dry methane group, which migrates also in the gases from the Lime Factory resort.

## Figures and Tables

**Table 1.** Regions and locations: characteristics.

Name	Type	Latitude (North)	Longitude (East)
Ghizela’s Cross Spa	drilling	44° 53′ 31.35″	22° 25′ 28.29″
7 Hot Spas’s Right	drilling	44° 53′ 24.14″	22° 25′ 25.70″
Scorillo Spa	drilling	44° 53′ 18.10″	22° 25′ 32.46″
Neptun Spa 1	drilling	44° 53′ 8.59″	22° 25′ 7.73″
Dragalina Spa	spring	44° 53′ 4.00″	22° 25′ 19.95″
Traian Spa	drilling	44° 52′ 52.99″	22° 24′ 57.68″
Decebal Spa	drilling	44° 52′ 34.99″	22° 24′ 49.99″
Well 5789	drilling	44° 52′ 27.87″	22° 25′ 0.08″
Lime Factory	drilling	44° 52′ 24.41″	22° 24′ 52.82″
Mehadia (coal mine)	–	44° 53′ 8.82″	22° 23′ 22.48"

**Table 2.** Gas composition of emanated gases from Herculane Spa area.

No	Location	Gas concentration (% volume)								[CH_4_]/[C_2_H_6_]
		CH_4_	N_2_	C_2_H_6_	Ar	O_2_	SH_2_	CO_2_	H_2_	
1	Ghizela’s Cross Spa	n.p.	82.62	n.p.	1.47	14.73	n.p.	1.18	n.p.	n.p.
2	7 Hot Spa’s Right	1.77	96.28	0.04	1.66	0.16	0.01	0.05	n.p.	44
3	Scorillo Spa	2.87	95.12	0.16	1.73	0.12	0.04	0.07	n.p.	18
4	Neptun Spa 1	60.47	35.41	0.81	0.57	0.02	0.41	0.30	1.72	75
5	Dragalina Spa	67.61	28.75	0.57	0.31	0.05	0.61	0.11	1.85	119
6	Traian Spa	64.12	31.20	0.68	0.40	0.06	0.81	0.38	1.76	94
7	Decebal Spa	70.88	25.54	0.84	0.47	0.04	0.15	0.08	1.98	84
8	Well 5789	64.77	32.13	0.60	0.36	0.02	0.01	0.02	1.68	108
9	Lime Factory	59.51	37.06	0.69	0.60	0.15	0.03	0.66	1.43	86
10	Mehadia (coal mine)	12.01	8.05	n.p.	0.17	n.p.	0.14	79.63	0.31	n.p.

n.p. = not present
